# SYTO dyes and EvaGreen outperform SYBR Green in real-time PCR

**DOI:** 10.1186/1756-0500-4-263

**Published:** 2011-07-28

**Authors:** Anne C Eischeid

**Affiliations:** 1 Division of Bioanalytical Chemistry, Office of Regulatory Science, Center for Food Safety and Applied Nutrition, U.S. Food and Drug Administration, 5100 Paint Branch Parkway, College Park, MD, USA 20740

## Abstract

**Background:**

Real-time PCR can be carried out using either probes or DNA dyes. SYBR Green has been used the most, but it suffers from several drawbacks. Numerous other DNA dyes are commercially available, but with limited structural information. Dye behavior in real time PCR is difficult to predict, so empirical data are needed. In the work described here, a panel of 23 different DNA dyes--including green, orange, and red SYTO dyes, EvaGreen, and SYBR Green--were evaluated with respect to their performance in real time PCR.

**Findings:**

Data were analyzed for reaction inhibition, effects on amplicon melting temperature, fluorescent signal strength, and reaction efficiency. This is the first report of reaction efficiency using alternatives to SYBR Green. Results indicated substantial variation in performance even within the SYTO dye family. EvaGreen and the SYTO dyes 13, 16, 80, and 82 performed better than SYBR Green in general, and high reaction efficiencies can be achieved using these dyes.

**Conclusions:**

Empirical data were generated for 23 DNA dyes. This paper confirms and extends previous findings that among commercially available DNA dyes, EvaGreen and certain SYTO dyes are the most desirable alternatives to the commonly used SYBR Green in real-time PCR.

## Background

Real-time PCR can be carried out using sequence-specific oligonucleotide probes or non-sequence specific DNA dyes [1,2]. Dyes offer greater flexibility and reduced cost, and they allow dissociation (melt) curve analysis of PCR products, while the most commonly used probes do not [2-4]. In cases where template sequence tends to vary, dye-based detection helps prevent false negatives that might result from basepair mismatches in a sequence-specific probe binding region [5,6]. Despite the advantages of dye-based detection and the wide variety of commercially available fluorescent DNA dyes, most real-time PCR has been conducted using SYBR Green. SYBR Green exhibits a very strong fluorescent signal, but it has been shown to inhibit the PCR reaction and has a narrow dynamic range and lower reproducibility than other detection chemistries [2,3,7]. Melt curve analysis using SYBR Green is complicated by the dye's effect on melting temperature and by dye redistribution which occurs during melting [4]. This occurs because SYBR Green must be used at low nonsaturating concentrations to prevent reaction inhibition [8-10].

A previous investigation of DNA dyes from several families suggested that SYTO dyes are the most promising for real-time PCR applications [7]. The SYTO dyes constitute a large family of commercially available cyanine dyes. Interaction of cyanine dyes with DNA is complex and is influenced by electrostatic, van der Waals, hydrophobic, and steric interactions, all of which are governed by the dye's chemical structure. Dye binding appears to be cooperative and is affected by dye concentration and dye-to-basepair ratio [11]. SYTO dyes are more hydrophobic than other cyanine dyes. They bind DNA based mainly on charge and primarily in the minor groove. Within the SYTO dye family, there are variations in fluorescent enhancement on nucleic acid binding, excitation and emission spectra, DNA/RNA selectivity, binding mode, and binding affinity (Invitrogen/Molecular Probes, probes.invitrogen.com). All of these characteristics may affect performance in PCR reactions. EvaGreen is another DNA dye which is less inhibitory to PCR than SYBR Green and is marketed as an alternative [12]. Detailed structural information is generally not available for individual dyes, making it difficult to predict their behavior in real-time PCR. Published work has not included data on fluorescent signal strength or investigation of reaction efficiency, a critical aspect of real-time PCR experiments [7,13]. The most significant advantage of real-time PCR over standard end-point PCR is that it can be used quantitatively; high reaction efficiencies are imperative for accurate quantitation [2,14]. Inhibitory dyes such as SYBR Green are likely to have adverse effects on reaction efficiency.

The difficulties associated with predicting a dye's performance in real-time PCR require that researchers rely on empirical data. Relatively few studies have been published on alternatives to SYBR Green; this is especially true for the SYTO dyes despite their promising performance [7,13]. In the work described here, EvaGreen and a panel of 21 green, orange, and red SYTO dyes were evaluated for their performance in real-time PCR and compared to the commonly used SYBR Green. Data were analyzed with respect to reaction inhibition, effects on amplicon melting temperature, fluorescent signal strength, and reaction efficiency. The objectives of this work were 1) to generate empirical data for a larger panel of SYTO dyes than has previously been reported and 2) to test reaction efficiency using the best-performing dyes. This work was conducted as part of a project focused on developing real-time PCR assays to detect shrimp, a crustacean food allergen.

## Results and Discussion

Optimal concentration for each dye was determined to be that which gave the best combination of low C_t_, high fluorescence, and low inhibition (Tables 1 and 2). C_t _values were lower in general for the mitochondrial 16S target (Table 1) than for the nuclear tropomyosin target (Table 2) because mitochondrial targets are present in higher copy number in cells. Increasing C_t _(or no C_t_) values at higher dye concentrations indicate that the dye inhibits the PCR reaction. Fluorescent signals were normalized to that of SYBR Green. Effects of SYBR Green on amplicon T_m _were determined using both fold (x) dye concentration--for comparison with EvaGreen data--and molar (μM) dye concentration--for comparison with SYTO dye data. In some cases, the dyes had very little effect on melting temperature. While they yielded low R^2 ^values, absolute differences in T_m _were small in these cases. This effect was more pronounced for the tropomyosin target (Table 2). No slope or R^2 ^values are reported for effects of SYTO 24 on T_m _because only the lowest concentration of dye yielded amplification. Effects of SYBR Green on T_m _were determined using experiments conducted with dye concentrations below 2 μM (1×).

**Table 1:** Reaction inhibition, fluorescent signal, and melting temperature data: 16S amplicon

*Dye *	*Optimal Concentration *	***C***_***t***_***: 0.64 μM*** *(0.32×)*	***C***_***t***_***: 2 μM*** *(1×)*	***C***_***t***_***: 10 μM*** *(5×)*	***C***_***t***_***: 20 μM*** *(10×)*	*Maximum Fluorescence*	***T***_***m ***_***shift*** ***slope (R***^***2***^***)***
SYBR Green*	0.64 μM (0.32×)	27.4 ± 0.78	no C_t _	no C_t_	no C_t_	1.00	5.89, × conc. 2.95, μM conc. (0.86)

EvaGreen*	5×	31.54 ± 0.21	29.98 ± 0.50	28.60 ± 0.50	not determined	1.03	0.50 (0.94)

Green*							

SYTO 11	2 μM	30.8 ± 1.26	29.9 ± 1.06	33.7 ± 2.58	no C_t_	0.83	0.41 (0.96)

SYTO 13	10 μM	31.8 ± 1.06	30.5 ± 1.16	29.1 ± 0.95	29.9 ± 1.11	0.85	0.15 (0.92)

SYTO 16	10 μM	29.8 ± 0.95	28.5 ± 0.92	27.7 ± 1.04	30.3 ± 1.47	0.99	0.17 (0.93)

SYTO 21	0.64 μM	28.5 ± 0.98	32.94 ± 3.7	no C_t_	no C_t_	0.79	1.73 (0.63)

SYTO 24	0.64 μM	31.0 ± 0.0	no C_t_	no C_t_	no C_t_	0.98	not determined

Orange*							

SYTO 80	20 μM	33.9 ± 0.57	32.4 ± 0.09	30.27 ± 0.01	29.4 ± 0.09	0.54	0.018 (0.33)

SYTO 81	20 μM	40.13 ± 0.0	35.7 ± 0.55	32.5 ± 0.10	32.0 ± 0.03	0.13	-0.002 (0.003)

SYTO 82	20 μM	31.8 ± 0.37	31.25 ± 0.06	29.4 ± 0.01	28.7 ± 0.08	0.77	0.040 (0.59)

SYTO 83	20 μM	39.6 ± 2.09	35.2 ± 0.77	32.7 ± 0.41	32.4 ± 0.17	0.12	0.029 (0.38)

Red**							

SYTO 17	20 μM	no C_t_	no C_t_	32.9	30.9	0.05	-0.001 (0.009)

SYTO 59	2 μM	28.9	28.0	28.5	37.2	0.29	0.16 (0.96)

SYTO 60	2 μM	29.5	29.37	no C_t_	no C_t_	0.16	0.24 (0.78)

SYTO 61	10 μM	32.0	29.86	27.76	31.55	0.17	0.06 (0.91)

SYTO 62	0.64 μM	28.8	30.75	no C_t_	no C_t_	0.18	0.26 (0.99)

SYTO 63	2 μM	28.9	28.6	43.0	no C_t_	0.20	0.12 (0.32)

SYTO 64	2 μM	34.8	31.7	31.7	34.0	0.15	0.012 (0.27)

No fluorescent signals were obtained for SYTO dyes 12, 14, 25, 84, or 85

*C_t _values are average ± SD for at 2-3 independent experiments

**C_t _values from one independent experiment, no SD

**Table 2:** Reaction inhibition, fluorescent signal, and melting temperature data: tropomyosin amplicon

*Dye *	*Optimal Concentration *	***C***_***t***_***: 0.64 μM*** *(0.32×)*	***C***_***t***_***: 2 μM*** *(1×)*	***C***_***t***_***: 10 μM*** *(5×)*	***C***_***t***_***: 20 μM*** *(10×)*	*Maximum Fluorescence*	***T***_***m ***_***shift*** ***slope (R***^***2***^***)***
SYBR Green*	0.64 μM	33.00 ± 0.10	35.07 ± 1.10	no C_t_	no Ct	1.00	3.99, × conc. 1.99, μM conc. (0.97)

EvaGreen*	5×	36.37 ± 0.48	35.04 ± 0.35	35.15 ± 1.13	not determined	0.91	0.36 (0.98)

Green*							

SYTO 11	2 μM	38.02 ± 0.97	36.42 ± 0.84	38.17 ± 1.03	42.27 ± 0	0.51	-0.20 (0.12)

SYTO 13	10 μM	37.58 ± 1.33	36.10 ± 1.32	36.06 ± 1.55	36.45 ± 2.06	0.87	0.001 (0.00)

SYTO 16	10 μM	37.00 ± 1.86	34.20 ± 1.05	34.04 ± 0.96	34.97 ± 0.53	1.07	0.030 (0.004)

SYTO 21	0.64 μM	34.55 ± 1.07	35.90 ± 0.83	no C_t_	no Ct	0.82	0.62 (0.03)

SYTO 24	0.64 μM	33.50 ± 0.01	no C_t_	no C_t_	no Ct	1.02	not determined

Orange*							

SYTO 80	20 μM	39.16 ± 2.44	37.33 ± 1.86	35.84 ± 0.02	34.78 ± 0.08	0.40	0.048 (0.008)

SYTO 81	20 μM	43.89 ± 0.10	40.62 ± 1.39	37.02 ± 0.39	36.45 ± 0.21	0.11	0.026 (0.002)

SYTO 82	20 μM	37.18 ± 1.85	35.59 ± 0.95	33.50 ± 0.11	33.44 ± 0.51	0.61	-0.17 (0.10)

SYTO 83	20 μM	42.20 ± 0.01	39.28 ± 0.99	37.04 ± 0.13	35.92 ± 0.93	0.10	0.037 (0.0051)

Red*							

SYTO 17	20 μM	no C_t_	42.26 ± 0.02	36.18 ± 0.14	35.32 ± 0.41	0.03	0.0009 (0.0005)

SYTO 59	10 μM	35.63 ± 0.09	35.15 ± 1.50	33.70 ± 0.10	35.53 ± 0.53	0.20	0.11 (0.83)

SYTO 60	2 μM	34.85 ± 0.41	34.29 ± 0.31	41.09 ± 1.97	no Ct	0.13	0.064 (0.10)

SYTO 61	10 μM	37.45 ± 1.68	35.38 ± 0.57	34.14 ± 0.98	35.00 ± 1.19	0.14	0.043 (0.58)

SYTO 62	0.64 μM	33.98 ± 0.42	33.69 ± 0.75	no C_t_	no Ct	0.17	0.035 (0.008)

SYTO 63	2 μM	34.78 ± 0.41	34.50 ± 0.31	37.07 ± 0.07	no Ct	0.12	0.24 (0.85)

SYTO 64	2 μM	36.22 ± 0.10	36.68 ± 1.62	36.31 ± 1.05	36.48 ± 0.95	0.05	-0.004 (0.013)

No fluorescent signals were obtained for SYTO dyes 12, 14, 25, 84, or 85

*C_t _values are average ± SD for at 2-3 independent experiments

There were significant differences in performance among the SYTO dyes. SYTO 21 and SYTO 24 were among the poorest-performing dyes in terms of reaction inhibition and effects on T_m_, while SYTO 13 and SYTO 16 were among the best. These are all green dyes in the same family and yet they exhibited significantly different behavior; this emphasizes the need for empirical data. The results presented here are in general agreement with previous work showing that SYTO 13 and SYTO 16 have little effect on amplicon melting temperature and are less inhibitory to PCR than SYBR Green, though optimal concentrations were lower in the previous study [7]. This work was conducted using a Stratagene instrument equipped with halogen lamp excitation and photomultiplier tube (PMT) detection, while previous work was conducted using a BioRad instrument equipped with light-emitting-diode (LED) excitation and photodiode detection [7]. Thus, similar results have been obtained in independent laboratories employing instruments from different manufacturers and with fundamentally different optics. Fluorescent signal strength is in part a reflection of how well dye spectra match instrument detection channels. In the current study, green SYTO dyes had the greatest fluorescent signal strength. In general, the green dye spectra matched instrument detection channels better than the orange SYTO dyes, and the red SYTO dye spectra were the most divergent from available detection channels (Additional File 1: Table S1). Red dyes also had the lowest fluorescent signals.

**Figure 1:**
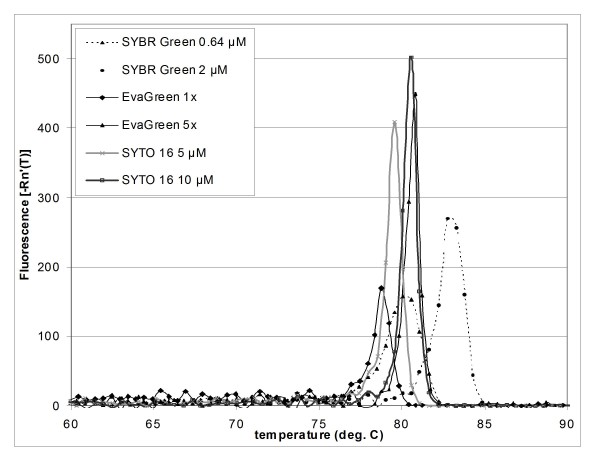
**Melt curves for SYBR Green, EvaGreen, and SYTO 16**. All reactions contained 1000 pg shrimp genomic DNA amplified using the 16S primer set. Similar data were obtained for all experiments and amounts of template DNA tested.

SYBR Green, EvaGreen and four SYTO dyes were used in tests of reaction efficiency for the 16S target. Two concentrations of each dye were tested (Table 3). Reaction efficiencies of 95%-101% and R^2 ^values of 0.98-0.99 were obtained for SYBR Green at 0.64 μM and for the other dyes at both concentrations tested. At 2 μM (1×) SYBR Green, the R^2 ^value was only 0.395, reflecting scatter and high variability in the data at this inhibitory dye concentration. Differences between reaction efficiency data (Table 3) and reaction inhibition data (Table 1) reported for SYBR Green are likely due to differences in thermal cycling, which was adjusted to optimize reaction efficiency. In order to achieve high reaction efficiencies, the annealing and extension step had to be carried out at a lower temperature for a longer time. This is in agreement with the findings of Hilscher et al. [15] who report that fast cycling times can result in lower reaction efficiencies. The work presented here demonstrates that high reaction efficiencies can be achieved using EvaGreen and the SYTO dyes 13, 16, 80, and 82 over a greater range of concentrations and at higher concentrations than SYBR Green. This is important not only for accurate and reliable quantitation, but also for melt curve analysis. Dyes which can be used at higher, saturating concentrations without adversely affecting the PCR reaction enable higher resolution melt curve analysis and even single-basepair mismatch discrimination [10,16]. SYTO 16 and EvaGreen gave higher, sharper peaks in melt curves than SYBR Green and the other dyes evaluated in this work (Figure 1). SYTO 16 displayed sharp melt curve peaks at both 5 μM and 10 μM, while peaks for EvaGreen were sharp at 5× but not at a 1× concentration.

**Table 3:** Reaction efficiency data (16S amplicon)

*Dye*	*Concentration*	***R***^***2***^	*Slope*	*Efficiency*
SYBR Green	0.32 × (0.64 μM)	0.985	-3.40	97%

SYBR Green	1× (2 μM)	0.395	-2.13	195%

EvaGreen	1×	0.995	-3.30	101%

EvaGreen	5×	0.999	-3.46	95%

SYTO 13	5 μM	0.993	-3.42	96%

SYTO 13	10 μM	0.993	-3.44	95%

SYTO 16	5 μM	0.983	-3.30	101%

SYTO 16	10 μM	0.992	-3.43	96%

SYTO 80	10 μM	0.992	-3.42	96%

SYTO 80	20 μM	0.986	-3.41	96%

SYTO 82	10 μM	0.998	-3.41	96%

SYTO 82	20 μM	0.996	-3.37	98%

## Conclusions

Real-time PCR data were generated using 21 SYTO dyes, EvaGreen and SYBR Green. Wide variation in performance within the same dye family emphasizes the need for empirical data of the type presented here. EvaGreen and the SYTO dyes 13, 16, 80, and 82 performed better than other dyes; EvaGreen and SYTO 16 produced the sharpest peaks in melt curve analysis.

## Methods

Farm-raised shrimp were obtained from a local market and DNA was extracted using the DNeasy Blood and Tissue Kit (Qiagen, Valencia, CA). Primers targeting the shrimp 16S rRNA gene (forward primer: TTGCGACCTCGATGTTGAATTAAGG, reverse primer: CCGGTCTGAACTCAGATCATGTAAGG, amplicon: TTGCGACCTCGATGTTGAATTAAGGGTTCCTTATAATGCAGCAGTTATAAAGGAGGGTCTGTTCGACCTTTAAATCCTTACATGATCTGAGTTCAGACCGG) were designed from conserved regions of the alignment provided in Khamnamtong et al. [17] and used to amplify a 101 bp fragment from the 3' end of this alignment. Primers targeting the shrimp tropomyosin gene (forward primer: TGCAGCAACTTGAGAACGACCTTG, reverse primer: TGTCCTTCTCCACAAGCTGGATGT, amplicon: TGCAGCAACTTGAGAACGACCTTGACCAGGTGCAGGAATCCTTGCTGAAGGCTA ACATCCAGCTTGTGGAGAAGGACA) were designed from Genbank accession number AY827100 using PrimerQuest (Integrated DNA Technologies, Coralville, IA). All primers were purchased from Integrated DNA Technologies. SYTO dyes were obtained from Invitrogen/Molecular Probes (Carlsbad, CA) and EvaGreen was obtained from Biotium (Hayward, CA). Molar concentrations of SYBR Green were calculated using the estimation provided by Zipper et al. [18]. PCR was carried out using the Brilliant SYBR Green QPCR Core Reagent Kit on an Mx3005P qPCR system supplied by Agilent Technologies (Santa Clara, CA). Reactions contained 1× PCR buffer, 3 mM MgCl_2_, 1.2 mM dNTP mix, 200 nM each primer, 3% DMSO, 4% glycerol, and 1.25 units Taq polymerase in 25 μl total volume. Data on reaction inhibition, fluorescent signal strength, and melting temperature for both the 16S target (Table 1) and the tropomyosin target (Table 2) were generated using 10 pg of shrimp genomic DNA per reaction. Thermal cycling for these data consisted of an initial step at 95°C for 10 minutes, and 45 cycles of denaturation at 95°C for 30 seconds plus annealing/extension at 65°C for 1 minute. Effects on amplicon melting temperature (T_m_) were determined by plotting T_m _vs. dye concentration. Slopes and R^2 ^values for T_m _data are reported for each dye (Tables 1 and 2). Reaction efficiency tests were carried out using the 16S gene target. Thermal cycling for reaction efficiency data (Table 3) consisted of an initial step at 95°C for 10 minutes, and 45 cycles of denaturation at 95°C for 30 seconds plus annealing/extension at 60°C for 2 minutes. Reaction efficiency data were generated by determining C_t _values using 10-fold dilutions of template DNA ranging from 0.1 to 1000 pg of shrimp genomic DNA per reaction. A linear standard curve of C_t _vs. log DNA concentration was plotted. Reaction efficiencies were calculated using the equation E = 10^(-1/m) ^-1, where E = reaction efficiency and m = slope of the linear standard curve [14].

## Competing interests

The author declares that they have no competing interests.

## Authors' contributions

AE conceived, designed, and carried out all experiments reported here and wrote the manuscript.

## Author Details

AE has over 14 years of molecular biology research experience and has conducted molecular biology and PCR work in both the life science and environmental engineering fields. She completed her Ph.D. at Duke University in 2009, and currently develops and validates real-time PCR assays for detection of food allergens at the U. S. Food and Drug Administration.

## Supplementary Material

Additional file 1 **Table S1**. Excitation and emission maxima of dyes and Mx3005P detection channels. Click here for file
