# Supplementary material for: SYTO dyes and EvaGreen outperform SYBR Green in real-time PCR
Source: BMC Res Notes. 2011 Jul 28;4:263. doi: 10.1186/1756-0500-4-263 (PMC3162529; doi:10.1186/1756-0500-4-263)
Supplement: Additional file 1 — Table S1. Excitation and emission maxima of dyes and Mx3005P detection channels. [file 1756-0500-4-263-S1.DOC]

**Supplementary Material**

**Table S1: Excitation and emission maxima of dyes and Mx3005P detection channels**

| **dye** | **dye excitation (nm)** | **dye emission (nm)** | **channel** | **channel excitation (nm)** | **channel emission (nm)** | **∆ excitation** | **∆ emission** |
| --- | --- | --- | --- | --- | --- | --- | --- |
| SYBR Green | 497 | 520 | SYBR | 492 | 516 | 5 | 4 |
| EvaGreen | 500 | 530 | SYBR | 492 | 516 | 8 | 14 |
|  |  |  |  |  |  |  |  |
| SYTO 11 | 508 | 527 | SYBR | 492 | 516 | 16 | 11 |
| SYTO 13 | 488 | 509 | SYBR | 492 | 516 | 4 | 7 |
| SYTO 16 | 488 | 518 | SYBR | 492 | 516 | 4 | 2 |
| SYTO 21 | 494 | 517 | SYBR | 492 | 516 | 2 | 1 |
| SYTO 24 | 490 | 515 | SYBR | 492 | 516 | 2 | 1 |
|  |  |  |  |  |  |  |  |
| SYTO 80 | 531 | 545 | HEX | 535 | 555 | 4 | 10 |
| SYTO 81 | 530 | 544 | HEX | 535 | 555 | 5 | 11 |
| SYTO 82 | 541 | 560 | Cy3 | 545 | 568 | 4 | 8 |
| SYTO 83 | 543 | 559 | Cy3 | 545 | 568 | 2 | 9 |
|  |  |  |  |  |  |  |  |
| SYTO 17 | 621 | 634 | Cy5 | 635 | 665 | 14 | 31 |
| SYTO 59 | 622 | 645 | Cy5 | 635 | 665 | 13 | 20 |
| SYTO 60 | 652 | 678 | Cy5 | 635 | 665 | 17 | 13 |
| SYTO 61 | 628 | 645 | Cy5 | 635 | 665 | 7 | 20 |
| SYTO 62 | 652 | 676 | Cy5 | 635 | 665 | 17 | 11 |
| SYTO 63 | 657 | 673 | Cy5 | 635 | 665 | 22 | 8 |
| SYTO 64 | 599 | 619 | ROX | 585 | 610 | 14 | 9 |
